# *In vitro* Photodynamic Therapy of Polymicrobial Biofilms Commonly Associated With Otitis Media

**DOI:** 10.3389/fmicb.2020.558482

**Published:** 2020-08-31

**Authors:** Kirsten L. Bair, Gal Shafirstein, Anthony A. Campagnari

**Affiliations:** ^1^Department of Microbiology and Immunology, Jacobs School of Medicine, University at Buffalo, State University of New York, Buffalo, NY, United States; ^2^Department of Cell Stress Biology, Photodynamic Therapy Center, Roswell Park Comprehensive Cancer Center, Buffalo, NY, United States; ^3^The Witebsky Center for Microbial Pathogenesis and Immunology, University at Buffalo, State University of New York, Buffalo, NY, United States

**Keywords:** photodynamic therapy, otitis media, biofilm, polymicrobial, *Moraxella catarrhalis*, *Streptococcus pneumoniae*, non-typeable *Haemophilus influenzae*

## Abstract

Otitis media (OM) is a prevalent pediatric infection characterized by painful inflammation of the middle ear. There are more than 700 million cases of OM diagnosed globally each year, with 50% of affected children under 5 years of age. Further, OM is the most common reason for children to receive antibiotic treatment in developed countries. The most recent work on this dynamic disease indicates that biofilms and polymicrobial infections play a role in recurrent OM and chronic OM, which are difficult to eradicate using standard antibiotic protocols. Antimicrobial photodynamic therapy (aPDT) is a promising new strategy for the treatment of resistant bacteria and persistent biofilms which lead to chronic infections. While PDT continues to be successfully used for oncological, dermatological, and dental applications, our work focuses on the efficacy of aPDT as it relates to otopathogens responsible for OM. Previous studies from our laboratory and others have shown that non-typeable *Haemophilus influenzae, Streptococcus pneumoniae* and *Moraxella catarrhalis*, the three most common otopathogens, are susceptible to different forms of aPDT. However, many cases of OM involve multiple bacteria and to date no one has investigated the efficacy of this technology on these complex polymicrobial biofilms. We treated polymicrobial biofilms of the three most common otopathogens with the photosensitizer Chlorin e6 (Ce6) and a continuous wave 405 ± 10 nm light emitted diode. Our data show significant bactericidal activity on polymicrobial biofilms associated with OM. These studies indicate that aPDT warrants further analysis as a possible treatment for OM and our results provide the foundation for future studies designed to identify the optimal aPDT parameters for polymicrobial biofilm-associated infections of the middle ear.

## Introduction

Otitis media (OM) is a common infection of the pediatric population. Approximately 50 to 80% of children in the US have experienced an episode of OM by 1 year of age, with peak incidence of disease in children 3 years of age (Murphy and Parameswaran, 2009). Recently, the multivalent pneumococcal conjugate vaccine and the *Haemophilus influenzae* type b vaccine have changed the landscape of nasopharyngeal colonization (Cohen et al., 2006; Revai et al., 2006; Dagan, 2009). Despite the success of these vaccines, non-typeable *Haemophilus influenzae* (NTHi), *Moraxella catarrhalis* and *Streptococcus pneumoniae* still account for approximately 95% of OM cases (Broides et al., 2009). Further, antibiotic resistance and decreased sensitivity to current treatment strategies is being reported among these otopathogens (Pichichero, 2000; Zielnik-Jurkiewicz and Bielicka, 2015; Sillanpaa et al., 2016; Korona-Glowniak et al., 2018). This is the result of polymicrobial biofilms associated with infection (Hall-Stoodley et al., 2006; Holder et al., 2015). Polymicrobial biofilms are difficult to treat due to poor drug penetration, conferred antibiotic protection and quiescent bacteria within biofilms. The lack of successful preventative and treatment strategies emphasizes the need for novel approaches to treat recurrent OM. Our work focuses on the efficacy of photodynamic therapy (PDT) with Chlorin e6 (Ce6) against polymicrobial otopathogen biofilms.

In PDT, the excitation of a light sensitive drug (photosensitizer) by visible light in the presence of oxygen induces the formation of singlet oxygen via the transfer of intermolecular energy, which ultimately results in cytotoxicity (Agostinis et al., 2011). PDT is currently being used as a cancer treatment and has recently shown promise for treating bacterial infections (reviewed in Dai et al., 2009; Cieplik et al., 2018; Mahmoudi et al., 2018). Antimicrobial PDT (aPDT) has several benefits as a therapeutic approach. aPDT is broadly bactericidal and anatomically site specific, subsequently limiting deleterious effects on the overall microbiome such as those seen with broad-spectrum antibiotics. Additionally, because the bactericidal mechanism of aPDT is based on non-selective oxidative damage to multiple, critical cellular biomolecules (e.g., proteins, lipids, and nucleic acids), it has been deemed unlikely bacteria could develop resistance (Maisch, 2009, 2015; Wainwright et al., 2017; Cieplik et al., 2018).

Previous studies from our lab and others have suggested that this technology could be effective vs. bacteria associated with OM (Jung et al., 2009; Luke-Marshall et al., 2014, 2020; Liu et al., 2020). We recently reported aPDT using Ce6 exhibits significant bactericidal activity against monomicrobial planktonic and biofilms of non-typeable *H. influenzae, S. pneumoniae* and *M. catarrhalis* (Luke-Marshall et al., 2020). However, it is now well accepted that many cases of recurrent and chronic OM involve multiple bacterial species in complex biofilms. In this study, we have identified an aPDT treatment that is effective against dual and triple species biofilms. To our knowledge, this is the first study to investigate the efficacy of aPDT on polymicrobial biofilms of the three major otopathogens associated with OM. Bacterial viability was significantly decreased when treated with Ce6 and excited with a 405 nm light emitted diode (LED) for 20 min to deliver 123.1 J/cm^2^ or in two sessions of 15 min (total 30 min) to deliver a total of 184.7 J/cm^2^. Further, it appears that polymicrobial biofilms are more resistant to our aPDT protocol as compared to monomicrobial biofilms. However, two consecutive treatments of polymicrobial biofilms at a minimal drug concentration, incubation time and LED exposure resulted in significant bactericidal activity, suggesting that aPDT warrants further analysis as a possible treatment strategy for recurrent or chronic OM.

## Materials and Methods

### Bacteria and Culture Conditions

*M. catarrhalis* strain 7169 is a clinical middle ear isolate previously described (Faden et al., 1997). Minimally passaged planktonic *M. catarrhalis* cultures were grown at 37°C, 180 RPM, aerobically in chemically defined minimal media (CDM). NTHi strain 86-028NP is a clinical isolate from a pediatric patient who underwent a tympanostomy for chronic OM (Kennedy et al., 2000). NTHi cultures were grow at 37°C, 180 RPM, aerobically in CDM. *S. pneumoniae* EF3030 is a serotype 19F otitis media isolate that was grown statically and anaerobically in CDM at 37°C (Andersson et al., 1983). *M. catarrhalis* 11-01-125, NTHi, 11-01-125 and *S. pneumoniae* 11-01-125 are recent clinical isolates from a child with OM. Each species was grown as outlined above and maintained for long term storage at -80°C.

### Photosensitizer

A > 98% pure Chlorin e6 trisodium salt (Ce6) in 30 mg vials (Fotolon^®^, Apocare Pharma GmbH, Bielefeld, Germany) was used in all the experiments. The Ce6 was dissolved in saline, at room temperature to a working concentration of 6 mg/mL. The solution was protected from light and used within 3–4 h at room temperature or kept at 4°C for 1–2 days. The Ce6, a derivative of chlorophyll, has a maximum absorption at 405 nm and a secondary absorption peak at 664 nm. The fluorescence emission peak is within the range of 600–760 nm.

### Light Source Parameters and Dosimetry

The bacteria were illuminated with a continuous wave 405 ± 10 nm light emitted diode (UHP-F5-405, Prizmatix, Givat Shmuel, Israel). The beam size was 6 cm in diameter and the total power was 2.9 W, resulting in 102.6 mW/cm^2^ at the treated surface. To evaluate if the response to aPDT was related to the Ce6 fluorescence intensity, we used a light dosimetry system to monitor the change of fluorescence intensity during treatment. The dosimetry system set up and calibration was previously described (Oakley et al., 2015; Shafirstein et al., 2016; Chamberlain et al., 2019; Luke-Marshall et al., 2020). We detected the fluorescence light with a calibrated isotropic light dosimetry fiber (IP85, Medlight SA, Ecublens, Switzerland) that was placed above (12 cm) the treated wells. The relative change in the fluorescence intensity was computed by measuring the change in the area under the curve of the fluorescence peak (640–740 nm), during treatment.

### Biofilm aPDT Treatment

Stationary *in vitro* biofilms were grown in 24-well plates on a monolayer of NCI-H292 bronchial carcinoma cells (ATCC CCL-1848) as previously described (Marks et al., 2012; Reddinger et al., 2016; Luke-Marshall et al., 2020). Planktonic cultures were grown to an OD_600_ of ∼0.2 (∼10^7^ CFU per mL) and diluted 1:100 prior to seeding. Wells received 350 μL of each culture as appropriate during seeding of either monomicrobial, dual species or triple species biofilms and CDM was added as necessary to obtain a final well volume of 1,050 μL. A total of two wells per treatment condition were seeded per assay and a minimum of three independent assays were completed. Biofilms were incubated statically at 34°C and 5% CO_2_. Media changes were completed at 4 and 20 h post-seeding by replacing all spent media with 1 mL fresh CDM. After 24 h of growth the media was removed and replaced with 1 mL saline. Freshly diluted Ce6 was immediately added to each well (final concentration of 10 or 100 μM) and following incubation in the dark (15 or 30 min) at 37°C in 5% CO_2_, the drug was removed and the biofilms were washed with 1 mL saline. Control samples were exposed to LED only, drug only, or neither LED nor drug (no treatment control). The biofilms were excited with 405 nm LED for 20 min to deliver 123.1 J/cm^2^ with a fluence rate of 102.6 mW/cm^2^. For dual-treatment experiments, the aPDT protocol was performed as described (Luke-Marshall et al., 2020). In brief, immediately following completion of the initial aPDT treatment as detailed above, dual-treatment biofilm wells were retreated by replacing the contents of each well with fresh saline, or saline plus 10 μM Ce6, followed by incubation for 15 min as above and LED exposure for 15 min (for a total combined illumination of 30 min at 184.7 J/cm^2^). After treatment, the biofilms were harvested by carefully removing the supernatant and any planktonic or loosely attached bacteria and resuspending the biofilm in fresh saline via physical disruption with a pipette tip. Dilution plating onto selective media was utilized for CFU enumeration of *M. catarrhalis* (Muller-Hinton agar + vancomycin at 3 μg per mL), *S. pneumoniae* (Blood agar + gentamycin at 4 μg per mL), and NTHi (Chocolate agar + clarithromycin at 2 μg per mL). Plates were incubated for 24 h at 37°C and 5% CO_2_.

### Statistical Analyses

Log transformed CFU per mL values were checked for normal distribution using the Shapiro-Wilk test. Normally distributed data sets were analyzed by one-way ANOVA with Dunnett’s multiple comparison test as compared to control biofilms that received no drug or LED treatment. Alternatively, data sets that did not pass the Shapiro-Wilk test were analyzed using the non-parametric Kruskal-Wallis with Dunn’s multiple comparison test. *P* ≤ 0.05 were considered significant and were derived using a 95% confidence interval. Statistical analyses were performed with Prism 8 from GraphPad Software, Inc. (La Jolla, CA).

## Results

### aPDT With Ce6 on Polymicrobial Biofilms

**FIGURE 1:**
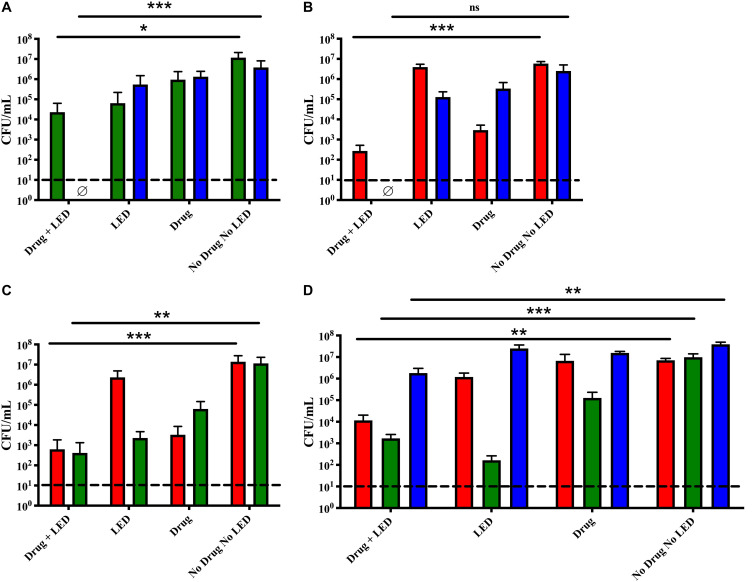
aPDT treatment of dual and triple species biofilms. Dual species biofilms of **(A)** *M. catarrhalis* (green) and NTHi (blue), **(B)** *S. pneumoniae* (red) and NTHi (blue), **(C)** *S. pneumoniae* (red) and *M. catarrhalis* (green), and triple species biofilms **(D)** were incubated for 15 min with 10 mM Ce6 followed by a 20 min 405 nm LED exposure. Surviving bacteria were quantitated by CFU enumeration. Bars represent the mean and standard deviation of three independent assays with a minimum of two technical replicates. Dashed lines indicate the limit of detection. ∅ indicates no detectable CFU were enumerated for the test condition. Statistically significant differences are denoted as *P < 0.05, **P < 0.01, ***P < 0.001; ns, not significant.

The viability of *M. catarrhalis* and NTHi were significantly reduced in dual species biofilms following the initial aPDT treatment (Figure 1A). While NTHi was completely killed, *M. catarrhalis* was reduced by approximately 3-logs as compared to the no treatment control. Both species showed some sensitivity to the Ce6 alone and the LED alone, as seen in the control wells. In addition, this aPDT method also elicited significant bactericidal activity toward NTHi and *S. pneumoniae* dual species biofilms (Figure 1B). NTHi was completely killed by the treatment and *S. pneumoniae* was reduced by approximately 4-logs. Of note, the statistical analysis was unable to account for the killing of NTHi by *S. pneumoniae* in the control, a previously published phenomena (Bair and Campagnari, 2020). For this reason, the difference in NTHi in the control vs. treated was not statistically significant despite the bacterial burden being reduced to below detectable levels. *S. pneumoniae* and *M. catarrhalis* dual species biofilms were similarly reduced to approximately 10^2^ CFU/mL each following treatment (Figure 1C). This was a significant reduction of nearly 4-logs and the remaining bacterial burden was slightly above detectable limits. Taken together the defined treatment parameters were effective at treating otopathogens in various dual species environments.

When the treatment was assessed vs. triple species biofilms, bactericidal activity against *M. catarrhalis* and *S. pneumoniae* resulted in 3-log and 2-log reductions, respectfully. Although NTHi was also significantly reduced, this bacterium appeared less sensitive to this aPDT approach, exhibiting a 1-log reduction (Figure 1D). Previous work by our lab described the stabilization of triple species biofilms by *M. catarrhalis*, which likely plays a factor in the decreased sensitivity of NTHi to treatment (Bair and Campagnari, 2020). In each case, the viability of the biofilm-associated bacteria was significantly reduced in each species as compared to controls. However, this aPDT approach needed adjustment to improve the bacterial reduction to more biologically relevant levels.

### Increased Ce6 Incubation Time

**FIGURE 2:**
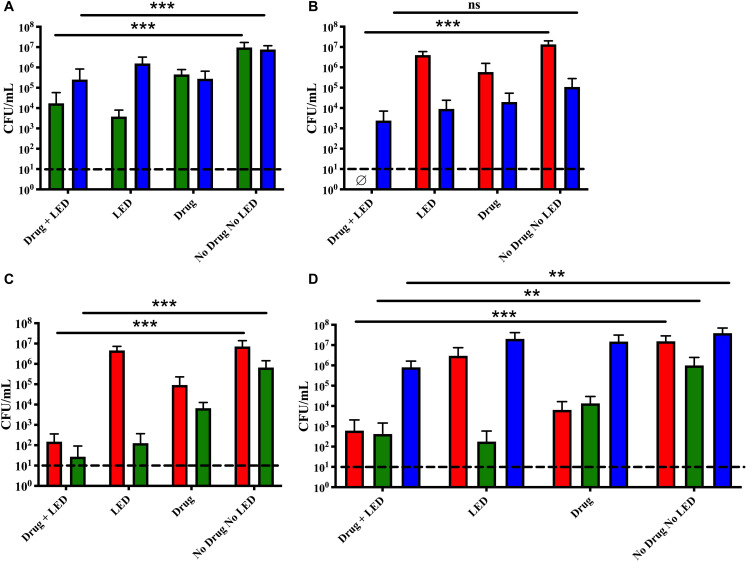
Extended drug incubation time of dual and triple species biofilms did not increase aPDT efficacy. Dual species biofilms of **(A)** *M. catarrhalis* (green) and NTHi (blue), **(B)** *S. pneumoniae* (red) and NTHi (blue), **(C)** *S. pneumoniae* (red) and *M. catarrhalis* (green), and triple species biofilms **(D)** were incubated for 30 min with 10 μM Ce6 followed by a 20 min 405 nm LED exposure. Surviving bacteria were quantitated by CFU enumeration. Bars represent the mean and standard deviation of three independent assays with a minimum of two technical replicates. Dashed lines indicate the limit of detection. ∅ indicates no detectable CFU were enumerated for the test condition. Statistically significant differences are denoted as **P < 0.01 and ***P < 0.001; ns, not significant.

It has been documented that Gram-negative bacteria are less susceptible to aPDT because of their cell wall structure (reviewed in Sperandio et al., 2013). To improve penetration of Ce6 through the biofilm structure and enhance interaction with the bacterial cell wall of NTHi and *M. catarrhalis*, we increased the drug incubation time to 30 min while leaving all other aPDT parameters the same as described (Figure 2A). The extended incubation with Ce6 did not improve the efficacy of the aPDT treatment for *M. catarrhalis* or NTHi in dual species, as compared to the previous treatment with an incubation time of 15 min (Figure 1A), as the viability of each species was reduced between 1 and 2 logs.

*S. pneumoniae* grown as a dual species biofilm with NTHi was completely eradicated below detectable levels (Figure 2B). Conversely, NTHi viability was reduced by approximately 1-log, as was seen in the other dual species conditions, suggesting that prolonged incubation with Ce6 did not increase bactericidal activity against NTHi. In contrast, dual species biofilms of *S. pneumoniae* and *M. catarrhalis* were significantly reduced by approximately 4–5 logs following the extended drug incubation (Figure 2C). These results are similar to those observed with the shorter incubation time (Figure 1C). Lastly, *S. pneumoniae* and *M. catarrhalis* were significantly reduced in triple species biofilms (Figure 2D). Although this treatment elicited statistically significant bactericidal effects on NTHi, the viability reduction was only 1-log. Of note, NTHi was less sensitive to treatment in each of the environments tested suggesting a longer incubation time did not improve the effectiveness of this aPDT treatment.

### Increased Ce6 Concentration

**FIGURE 3:**
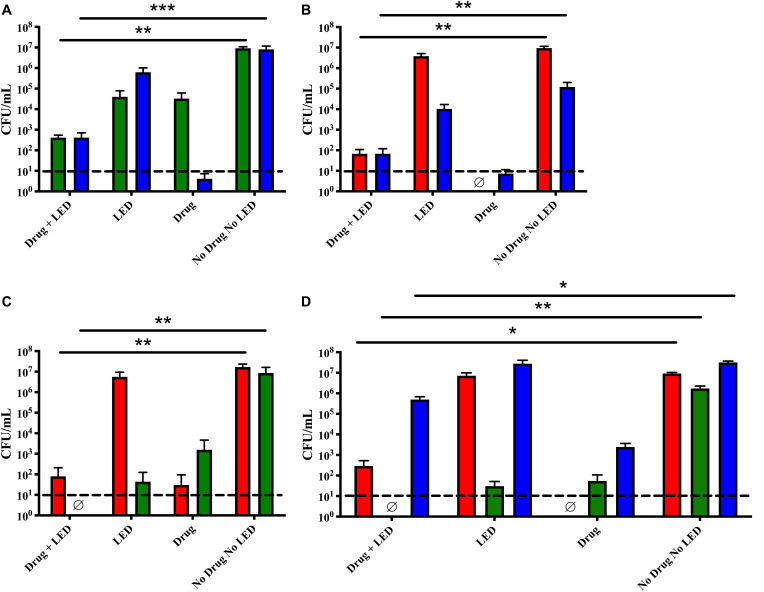
Increased drug concentration did not increase aPDT efficacy and dark toxicity was observed. Dual species biofilms of **(A)** *M. catarrhalis* (green) and NTHi (blue), **(B)** *S. pneumoniae* (red) and NTHi (blue), **(C)** *S. pneumoniae* (red) and *M. catarrhalis* (green), and triple species biofilms **(D)** were incubated for 30 min with 100 μM Ce6 followed by a 20 min 405 nm LED exposure. Surviving bacteria were quantitated by CFU enumeration. Bars represent the mean and standard deviation of three independent assays with a minimum of two technical replicates. Dashed lines indicate the limit of detection. ∅ indicates no detectable CFU were enumerated for the test condition. Statistically significant differences are denoted as *P < 0.05, **P < 0.01, ***P < 0.001.

We further expanded our treatment parameters by increasing the Ce6 concentration to 100 μM to insure minimum inhibitory concentration was being achieved. As above, all other aPDT parameters remained constant. *M. catarrhalis* and NTHi in dual species biofilms were each reduced 5-logs by treatment with the increased Ce6 concentration (Figure 3A). NTHi was sensitive to Ce6 alone at a concentration of 100 μM. In addition, NTHi and *S. pneumoniae* in dual species were significantly reduced by Ce6 and LED treatment as well as the drug only control (Figure 3B). The same was true for *S. pneumoniae* and *M. catarrhalis* in dual species (Figure 3C) as the aforementioned treatment conditions with an increased Ce6 concentrations completely eradicated the *M. catarrhalis* biofilm to below detectable limits and *S. pneumoniae* biofilm was reduced by 5 logs compared to the no treatment control. As seen with the other treatment conditions, *S. pneumoniae* and *M. catarrhalis* were significantly reduced in triple species biofilms, while NTHi was only reduced by 1-log (Figure 3D).

### Successive aPDT Treatments

**FIGURE 4:**
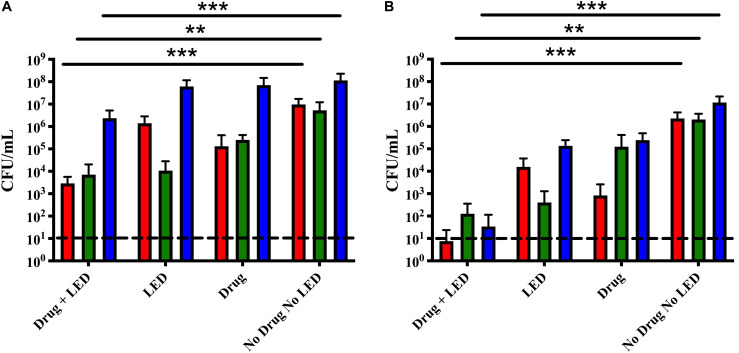
Successive aPDT treatments of dual and triple species biofilms improved treatment efficacy. **(A)** Single treatment. Biofilms were incubated for 15 min with 10 μM Ce6 followed by a 20 min 405 nm LED exposure. **(B)** Dual treatment. Biofilms were treated twice using the above parameters in two sequential cycles. Surviving biofilm-associated *S. pneumoniae* (red), *M. catarrhalis* (green), and NTHi (blue) were quantified by CFU enumeration. Dashed lines indicate the limit of detection. Statistically significant differences are denoted as **P < 0.01 and ***P < 0.001.

Based on our recently published data, we tested the successive dual-treatment approach on polymicrobial otopathogen biofilms under the hypothesis that the first round of treatment weakens the integrity of the biofilm structure so that the second treatment would be more effective at killing the remaining bacteria (Luke-Marshall et al., 2020). Triple species biofilms were treated once or twice with 10 μM Ce6 for 15 min followed by a 15 min LED exposure. A single treatment successfully reduced *S. pneumoniae* and *M. catarrhalis* 3-logs as compared to the no treatment control (Figure 4A). While statistically significant, NTHi was again only reduced by 1-log. After a second treatment, the remaining bacterial burden of all three species was significantly reduced to at or below 10^2^ CFU (Figure 4B). These data indicate the dual treatment protocol significantly reduced overall bacterial burden of all three otopathogen species, in comparison to a single treatment. We repeated these studies using the same aPDT parameters on triple species biofilms of recent clinical isolates obtained from a child with otitis media. The results of these dual-treatment studies showed significant bactericidal activity against *M. catarrhalis* 11-01-125 (99.9% kill rate), NTHI 11-01-125 (99.3% kill rate), and *S. pneumoniae* 11-01-125 (99.9% kill rate) confirming our approach is not strain specific (data not shown). Further, we did not observe any photobleaching following a single treatment or two successive treatments suggesting that the optimal deliverable dose was achieved (data not shown).

## Discussion

The decreased efficacy of antibiotics and other antimicrobials has become a global health problem and the implications of this reach into many common infections, reinforcing the need for novel treatment approaches. Herein we describe the use of aPDT with Ce6 as a treatment for polymicrobial biofilms such as those commonly associated with OM. OM is a major health problem in the pediatric population and is the primary cause for office visits and antibiotic treatment in young children. It is now widely accepted that chronic or recurrent bacterial OM is caused by biofilms composed of three primary otopathogens and is frequently polymicrobial in nature. Previous studies from our laboratory have shown that planktonic and biofilm-associated *M. catarrhalis, S. pneumoniae* and NTHi were susceptible to aPDT with Ce6 (Luke-Marshall et al., 2020). These studies further showed that two successive aPDT treatments on established bacterial biofilms significantly improved the efficacy of the treatment. While these studies were very important in demonstrating the potential use of aPDT in the treatment of the most common otopathogens, they were focused on single species biofilms, consistent with a monomicrobial infection. Given that many episodes of OM are complicated by the presence of multiple bacterial species we evaluated the efficacy of aPDT against these otopathogens in several different polymicrobial environments.

Our initial treatment parameters of 10 μM Ce6 with an incubation time of 15 min followed by 20 min of LED exposure significantly reduced the viability of the biofilms in dual species combinations. In addition, biofilms consisting of all three bacterial species decreased following treatment with these same parameters. However, while these reductions were statistically significant, the post-treatment bacterial burden remained fairly high (∼10^4^ to 10^6^ CFU) indicating that these aPDT parameters had to be modified in order to maximize bactericidal activity.

We initially increased the drug incubation time; however, that did not significantly improve the treatment efficacy. These results showed a level of bacterial kill consistent with the previous data and the remaining bacterial burden was comparable in all environments. Our next modification was to increase the Ce6 concentration with the caveat that excess photosensitizer could result in photosensitizer aggregation that decreases photoactivity and adversely affect our results (Tada and Baptista, 2015). aPDT treatment with 100 μM Ce6 significantly reduced the viability of all species in polymicrobial biofilms. The remaining bacterial burden was low in all environments with the exception of NTHi in the triple species biofilm, which was significantly reduced as compared to the no treatment control, however approximately 10^5^ CFU/mL remained following these treatment parameters. The LED only control was similar among conditions for all 20 min treatments, resulting in a slight reduction of each bacterial species. This is likely the result of activation of endogenous porphyrins generating ROS stress (Lanzilotto et al., 2018; Tsolekile et al., 2019). *M. catarrhalis* was the most susceptible to LED treatment, consistent with recent studies assessing the effects of blue light on *M. catarrhalis* monomicrobial biofilms (Liu et al., 2020). Of note, there was cytotoxicity of Ce6 alone at 100 μM. Ideal treatment parameters should have minimal dark toxicity and have additive bactericidal effects when combining treatment with Ce6 and LED exposure. Our results suggest treatment at 100 μM exceeded the minimum concentration required and treated bacteria with toxic levels of photosensitizer. Susceptibility to photosensitizer alone was different among bacterial species and future considerations should be made when defining ideal treatment conditions for all polymicrobial conditions.

Previous work from our lab has characterized the stability of triple species biofilms with these otopathogens (Bair and Campagnari, 2020). Membrane permeability has been a major variable in the success of aPDT against Gram-negative vs. Gram-positive bacteria and several studies have explored the use of membrane disorganizing agents in combination with PDT (Hamblin et al., 2002; Tegos et al., 2006). Although our original treatment parameters for polymicrobial biofilms were not optimal, our results did show significant bactericidal activity vs. *M. catarrhalis*, *S. pneumoniae* and to a lesser degree, NTHi. Based on these results and our recent data demonstrating that two successive treatments of aPDT significantly increased the bactericidal effects on monomicrobial biofilms, we hypothesized that the first round of aPDT treatment could weaken the integrity of the triple species biofilm structure which could make two successive treatments more effective at killing polymicrobial biofilms (Luke-Marshall et al., 2020). This experimental strategy proved to be extremely encouraging as two successive treatments of aPDT using the same parameters elicited significant bactericidal activity against these complex polymicrobial biofilms. All three otopathogens in these biofilms exhibited a ∼4–5 log reduction in viability decreasing to a final count of 10^2^ CFU/ml or lower (corresponding to a >99.9% kill rate), indicating this treatment approach elicited both statistically and biologically relevant reductions to the overall bacterial burden.

aPDT has many features that suggest this methodology has potential as a novel therapeutic treatment, particularly for chronic or recurrent OM. The ear canal and tympanic membrane are readily accessible to a LED or other light source and photosensitizer can easily be introduced into the middle ear via myringotomy or tympanostomy tubes. In addition, the middle ear cavity is quite small, which likely maximize the contact of photosensitizer with the bacterial biofilms while minimizing diffusion from the target. Moreover, the development of resistance to aPDT is highly improbable because the mechanism of action differs significantly from antibiotics. Whereas antibiotics act on very specific and limited targets, aPDT produces unrepairable damage in many different bacterial biomolecules that are essential for survival, resulting in rapid cell death (Maisch, 2009, 2015; Wainwright et al., 2017; Cieplik et al., 2018). More recently, an extensive study that included multiple species of antibiotic resistant bacterial and fungal pathogens, showed that all species evaluated were highly sensitive to the bacterial activity of aPDT and these authors concluded that resistance to aPDT must be considered highly unlikely (Sabino et al., 2020). However, there have been multiple reports describing an increase in resistance to oxidative stress in response to sublethal doses of aPDT, so it seems that currently there is no universal agreement as to whether bacteria have the potential to develop aPDT resistance (reviewed in Kashef and Hamblin, 2017; Cieplik et al., 2018).

While far more studies are needed, the significant reduction in the biofilm burden resulting from our aPDT methodology is physiologically relevant, as it is possible that the host immune response in combination with the appropriate antibiotics could ultimately clear the remaining bacteria. These promising results provide the foundation for future *ex vivo* and *in vivo* studies designed to determine if aPDT with Ce6 has potential as an effective treatment for chronic or recurrent OM, including infections caused by polymicrobial communities.

## Data Availability Statement

The raw data supporting the conclusions of this article will be made available by the authors, without undue reservation, to any qualified researcher.

## Author Contributions

KB performed all the aforementioned experimentation. GS provided scientific advice in the performance and analysis of the aPDT and light dosimetry, and contributed to the writing of the manuscript. KB and AC contributed to the conception of experimental design and preparation of the manuscript. All authors contributed to the article and approved the submitted version.

## Conflict of Interest

GS was a co-inventor in patent applications and technology owned by Roswell Park Comprehensive Cancer Center that licensed the use of the light dosimetry system in the field of photodynamic therapy. GS acknowledges a research grant support from Apocare Pharma GmbH that is not related to this particular study and was contracted to Roswell Park. The remaining authors declare that the research was conducted in the absence of any commercial or financial relationships that could be construed as a potential conflict of interest.

## References

[B1] Agostinis P. Berg K. Cengel K. A. Foster T. H. Girotti A. W. Gollnick S. O. (2011). Photodynamic therapy of cancer: an update. *CA Cancer J. Clin.* 61 250–281. 10.3322/caac.20114 21617154 PMC3209659

[B2] Andersson B. Dahmen J. Frejd T. Leffler H. Magnusson G. Noori G. (1983). Identification of an active disaccharide unit of a glycoconjugate receptor for pneumococci attaching to human pharyngeal epithelial cells. *J. Exp. Med.* 158 559–570. 10.1084/jem.158.2.559 6886624 PMC2187347

[B3] Bair K. L. Campagnari A. A. (2020). *Moraxella catarrhalis* promotes stable polymicrobial biofilms with the major otopathogens. *Front. Microbiol.* 10:3006. 10.3389/fmicb.2019.03006 32010085 PMC6974515

[B4] Broides A. Dagan R. Greenberg D. Givon-Lavi N. Leibovitz E. (2009). Acute otitis media caused by *Moraxella catarrhalis*: epidemiologic and clinical characteristics. *Clin. Infect. Dis.* 49 1641–1647. 10.1086/647933 19886799

[B5] Chamberlain S. Bellnier D. Yendamuri S. Lindenmann J. Demmy T. Nwogu C. (2019). An optical surface applicator for intraoperative photodynamic therapy. *Lasers Surg. Med.* 52 523–529. 10.1002/lsm.23168 31587314 PMC7131890

[B6] Cieplik F. Deng D. Crielaard W. Buchalla W. Hellwig E. Al-Ahmad A. (2018). Antimicrobial photodynamic therapy - what we know and what we don’t. *Crit. Rev. Microbiol.* 44 571–589. 10.1080/1040841x.2018.1467876 29749263

[B7] Cohen R. Levy C. de La Rocque F. Gelbert N. Wollner A. Fritzell B. (2006). Impact of pneumococcal conjugate vaccine and of reduction of antibiotic use on nasopharyngeal carriage of nonsusceptible pneumococci in children with acute otitis media. *Pediatr. Infect. Dis. J.* 25 1001–1007. 10.1097/01.inf.0000243163.85163.a8 17072121

[B8] Dagan R. (2009). Impact of pneumococcal conjugate vaccine on infections caused by antibiotic-resistant *Streptococcus pneumoniae*. *Clin. Microbiol. Infect.* 15(Suppl. 3), 16–20. 10.1111/j.1469-0691.2009.02726.x 19366365

[B9] Dai T. Huang Y. Y. Hamblin M. R. (2009). Photodynamic therapy for localized infections–state of the art. *Photodiagn. Photodyn. Ther.* 6 170–188. 10.1016/j.pdpdt.2009.10.008 19932449 PMC2811240

[B10] Faden H. Duffy L. Wasielewski R. Wolf J. Krystofik D. Tung Y. (1997). Relationship between nasopharyngeal colonization and the development of otitis media in children. Tonawanda/williamsville pediatrics. *J. Infect. Dis.* 175 1440–1445. 10.1086/516477 9180184

[B11] Hall-Stoodley L. Hu F. Z. Gieseke A. Nistico L. Nguyen D. Hayes J. (2006). Direct detection of bacterial biofilms on the middle-ear mucosa of children with chronic otitis media. *JAMA* 296 202–211. 10.1001/jama.296.2.202 16835426 PMC1885379

[B12] Hamblin M. R. O’Donnell D. A. Murthy N. Rajagopalan K. Michaud N. Sherwood M. E. (2002). Polycationic photosensitizer conjugates: effects of chain length and Gram classification on the photodynamic inactivation of bacteria. *J. Antimicrob. Chemother.* 49 941–951. 10.1093/jac/dkf053 12039886

[B13] Holder R. C. Kirse D. J. Evans A. K. Whigham A. S. Peters T. R. Poehling K. A. (2015). Otopathogens detected in middle ear fluid obtained during tympanostomy tube insertion: contrasting purulent and non-purulent effusions. *PLoS One* 10:e0128606. 10.1371/journal.pone.0128606 26039250 PMC4454603

[B14] Jung J. Y. Kwon P. S. Ahn J. C. Ge R. Suh M. W. Rhee C. K. (2009). In vitro and in vivo photodynamic therapy of otitis media in gerbils. *Laryngoscope* 119 1781–1787. 10.1002/lary.20568 19572273

[B15] Kashef N. Hamblin M. R. (2017). Can microbial cells develop resistance to oxidative stress in antimicrobial photodynamic inactivation? *Drug Resist Updat.* 31 31–42. 10.1016/j.drup.2017.07.003 28867242 PMC5673603

[B16] Kennedy B. J. Novotny L. A. Jurcisek J. A. Lobet Y. Bakaletz L. O. (2000). Passive transfer of antiserum specific for immunogens derived from a nontypeable *Haemophilus influenzae* adhesin and lipoprotein D prevents otitis media after heterologous challenge. *Infect. Immun.* 68 2756–2765. 10.1128/iai.68.5.2756-2765.2000 10768970 PMC97485

[B17] Korona-Glowniak I. Zychowski P. Siwiec R. Mazur E. Niedzielska G. Malm A. (2018). Resistant *Streptococcus pneumoniae* strains in children with acute otitis media- high risk of persistent colonization after treatment. *BMC Infect. Dis.* 18:478. 10.1186/s12879-018-3398-9 30253754 PMC6156860

[B18] Lanzilotto A. Kyropoulou M. Constable E. C. Housecroft C. E. Meier W. P. Palivan C. G. (2018). Porphyrin-polymer nanocompartments: singlet oxygen generation and antimicrobial activity. *J. Biol. Inorg. Chem.* 23 109–122. 10.1007/s00775-017-1514-8 29218642 PMC5756573

[B19] Liu X. Chang Q. Ferrer-Espada R. Leanse L. G. Goh X. S. Wang X. (2020). Photoinactivation of *Moraxella catarrhalis* using 405-nm blue light: implications for the treatment of otitis media. *Photochem. Photobiol.* 96 611–617. 10.1111/php.13241 32105346 PMC10125262

[B20] Luke-Marshall N. R. Hansen L. A. Shafirstein G. Campagnari A. A. (2020). Antimicrobial photodynamic therapy with chlorin e6 is bactericidal against biofilms of the primary human otopathogens. *mSphere* 5:e00492-20. 10.1128/mSphere.00492-20 PMC7364218 32669474

[B21] Luke-Marshall N. R. Mang T. S. Hansen L. A. Campagnari A. A. (2014). *Moraxella catarrhalis* is susceptible to antimicrobial photodynamic therapy with Photofrin. *Lasers Surg. Med.* 46 712–717. 10.1002/lsm.22287 25154610

[B22] Mahmoudi H. Bahador A. Pourhajibagher M. Alikhani M. Y. (2018). Antimicrobial photodynamic therapy: an effective alternative approach to control bacterial infections. *J. Lasers Med. Sci.* 9 154–160. 10.15171/jlms.2018.29 30809325 PMC6378356

[B23] Maisch T. (2009). A new strategy to destroy antibiotic resistant microorganisms: antimicrobial photodynamic treatment. *Mini. Rev. Med. Chem.* 9 974–983. 10.2174/138955709788681582 19601890

[B24] Maisch T. (2015). Resistance to antimicrobial photodynamic inactivatiof of bacteria. *Photochem. Photobiol. Sci.* 14 1518–1526. 10.1039/c5pp00037h 26098395

[B25] Marks L. R. Parameswaran G. I. Hakansson A. P. (2012). Pneumococcal interactions with epithelial cells are crucial for optimal biofilm formation and colonization *In Vitro* and *In Vivo*. *Infect. Immun.* 80 2744–2760. 10.1128/iai.00488-12 22645283 PMC3434590

[B26] Murphy T. F. Parameswaran G. I. (2009). *Moraxella catarrhalis*, a human respiratory tract pathogen. *Clin. Infect. Dis.* 49 124–131. 10.1086/599375 19480579

[B27] Oakley E. Wrazen B. Bellnier D. A. Syed Y. Arshad H. Shafirstein G. (2015). A new finite element approach for near real-time simulation of light propagation in locally advanced head and neck tumors. *Lasers Surg. Med.* 47 60–67. 10.1002/lsm.22313 25559426 PMC4304874

[B28] Pichichero M. E. (2000). Acute otitis media: part II. Treatment in an era of increasing antibiotic resistance. *Am. Fam. Phys.* 61 2410–2416. 10794582

[B29] Reddinger R. M. Luke-Marshall N. R. Hakansson A. P. Campagnari A. A. (2016). Host physiologic changes induced by influenza a virus lead to *Satphyolococcus aureus* biofilm dispersion and transition from asymptomatic colonizatio to invasive disease. *mBio* 7:e01235-16. 10.1128/mBio.01235-16 27507829 PMC4981728

[B30] Revai K. McCormick D. P. Patel J. Grady J. J. Saeed K. Chonmaitree T. (2006). Effect of pneumococcal conjugate vaccine on nasopharyngeal bacterial colonization during acute otitis media. *Pediatrics* 117 1823–1829. 10.1542/peds.2005-1983 16651345

[B31] Sabino C. P. Wainright M. Ribeiro M. S. Sellera F. P. dos Anjos C. Baptista M. S. (2020). Global priority multidrug-resistant pathogens do not resist photodynamic therapy. *J. Photochem. Photobiol.* 208:111893. 10.1016/j.jphotobiol.2020.111893 32446039

[B32] Shafirstein G. Battoo A. Harris K. Baumann H. Gollnick S. O. Lindenmann J. (2016). Photodynamic therapy of non-small cell lung cancer. Narrative review and future directions. *Ann. Am. Thorac. Soc.* 13 265–275. 10.1513/AnnalsATS.201509-650FR 26646726 PMC5015713

[B33] Sillanpaa S. Sipila M. Hyoty H. Rautiainen M. Laranne J. (2016). Antibiotic resistance in pathogens causing acute otitis media in Finnish children. *Int. J. Pediatr. Otorhinolaryngol.* 85 91–94. 10.1016/j.ijporl.2016.03.037 27240503

[B34] Sperandio F. F. Huang Y. Y. Hamblin M. R. (2013). Antimicrobial photodynamic therapy to kill Gram-negative bacteria. *Recent Pat. Antiinfect. Drug Discov.* 8 108–120. 10.2174/1574891x113089990012 23550545 PMC3740068

[B35] Tada D. B. Baptista M. S. (2015). Photosensitizing nanoparticles and the modulation of ROS generation. *Front. Chem.* 3:33. 10.3389/fchem.2015.00033 26075198 PMC4444965

[B36] Tegos G. P. Anbe M. Yang C. Demidova T. N. Satti M. Mroz P. (2006). Protease-stable polycationic photosensitizer conjugates between polyethyleneimine and chlorin(e6) for broad-spectrum antimicrobial photoinactivation. *Antimicrob. Agents Chemother.* 50 1402–1410. 10.1128/aac.50.4.1402-1410.2006 16569858 PMC1426948

[B37] Tsolekile N. Nelana S. Oluwafemi O. S. (2019). Porphyrin as diagnostic and therapeutic agent. *Molecules* 24:669. 10.3390/molecules24142669 31340553 PMC6680575

[B38] Wainwright M. Maisch T. Nonell S. Plaetzer K. Almeida A. Tegos G. P. (2017). Photoantimicrobials-are we afraid of light? *Lancet Infect. Dis.* 17 e49–e55. 27884621 10.1016/S1473-3099(16)30268-7 PMC5280084

[B39] Zielnik-Jurkiewicz B. Bielicka A. (2015). Antibiotic resistance of *Streptococcus pneumoniae* in children with acute otitis media treatment failure. *Int. J. Pediatr. Otorhinolaryngol.* 79 2129–2133. 10.1016/j.ijporl.2015.09.030 26454530

